# An environmental gradient dominates ecological and genetic differentiation of marine invertebrates between the North and Baltic Sea

**DOI:** 10.1002/ece3.8868

**Published:** 2022-05-20

**Authors:** Jonas C. Geburzi, Nele Heuer, Lena Homberger, Jana Kabus, Zoe Moesges, Kira Ovenbeck, Dirk Brandis, Christine Ewers

**Affiliations:** ^1^ Mangrove Ecology Leibniz Centre for Tropical Marine Research (ZMT) Bremen Germany; ^2^ 9179 Department of Organismic and Evolutionary Biology Museum of Comparative Zoology Harvard University Cambridge Massachusetts USA; ^3^ 9179 Zoological Museum Kiel University Kiel Germany; ^4^ 9179 Department Aquatic Ecotoxicology Institute of Ecology Diversity and Evolution Goethe University Frankfurt am Main Frankfurt am Main Germany

**Keywords:** adaptation, Baltic Sea, cytochrome oxidase, genetic differentiation, marine invertebrates, North Sea, population genetics, salinity

## Abstract

Environmental gradients have emerged as important barriers to structuring populations and species distributions. We set out to test whether the strong salinity gradient from the marine North Sea to the brackish Baltic Sea in northern Europe represents an ecological and genetic break, and to identify life history traits that correlate with the strength of this break. We accumulated mitochondrial cytochrome oxidase subunit 1 sequence data, and data on the distribution, salinity tolerance, and life history for 28 species belonging to the Cnidaria, Crustacea, Echinodermata, Mollusca, Polychaeta, and Gastrotricha. We included seven non‐native species covering a broad range of times since introduction, in order to gain insight into the pace of adaptation and differentiation. We calculated measures of genetic diversity and differentiation across the environmental gradient, coalescent times, and migration rates between North and Baltic Sea populations, and analyzed correlations between genetic and life history data. The majority of investigated species is either genetically differentiated and/or adapted to the lower salinity conditions of the Baltic Sea. Species exhibiting population structure have a range of patterns of genetic diversity in comparison with the North Sea, from lower in the Baltic Sea to higher in the Baltic Sea, or equally diverse in North and Baltic Sea. Two of the non‐native species showed signs of genetic differentiation, their times since introduction to the Baltic Sea being about 80 and >700 years, respectively. Our results indicate that the transition from North Sea to Baltic Sea represents a genetic and ecological break: The diversity of genetic patterns points toward independent trajectories in the Baltic compared with the North Sea, and ecological differences with regard to salinity tolerance are common. The North Sea–Baltic Sea region provides a unique setting to study evolutionary adaptation during colonization processes at different stages by jointly considering native and non‐native species.

## Graphical abstract

Environmental gradients have emerged as important barriers to structuring populations and species distributions. Using ecological and genetic data, we show that marine invertebrates exhibit significant structure across a strong salinity gradient from marine to brackish water. The length of the planktonic larval phase may be the sticking point for the colonization of brackish environments, as species with long‐lived planktonic larvae did not occur at lower salinities.

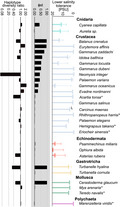

## 1 INTRODUCTION

Environmental gradients have emerged as important barriers to gene flow, structuring populations, and species distributions. The environment may be particularly important in the marine realm, where impenetrable barriers, such as landmasses, are relatively rare (Blanco‐Bercial et al., 2011; Ewers‐Saucedo & Wares, 2020). In particular, temperature (Ewers‐Saucedo et al., 2016), salinity (Sjöqvist et al., 2015), and water depth (Prada & Hellberg, 2021) may result in differentially adapted populations with limited gene flow between them. In addition to barriers to gene flow driven by the environment, other barriers to dispersal have been identified. Currents and upwelling limit dispersal for benthic invertebrates with a planktonic larval phase, albeit these barriers are not universal (Haye et al., 2014; Kelly & Palumbi, 2010; Wares et al., 2001). Just as important are stretches of unsuitable habitat, for example, long sandy beaches for rocky shore specialists (Ayre et al., 2009; Wares, 2019). In lieu of a planktonic phase or other long‐distance dispersal mechanisms, small‐scale population structure is commonplace in benthic species (Ewers‐Saucedo & Wares, 2020; Haye et al., 2014; Kyle & Boulding, 2000; Palumbi, 1994).

**FIGURE 1:**
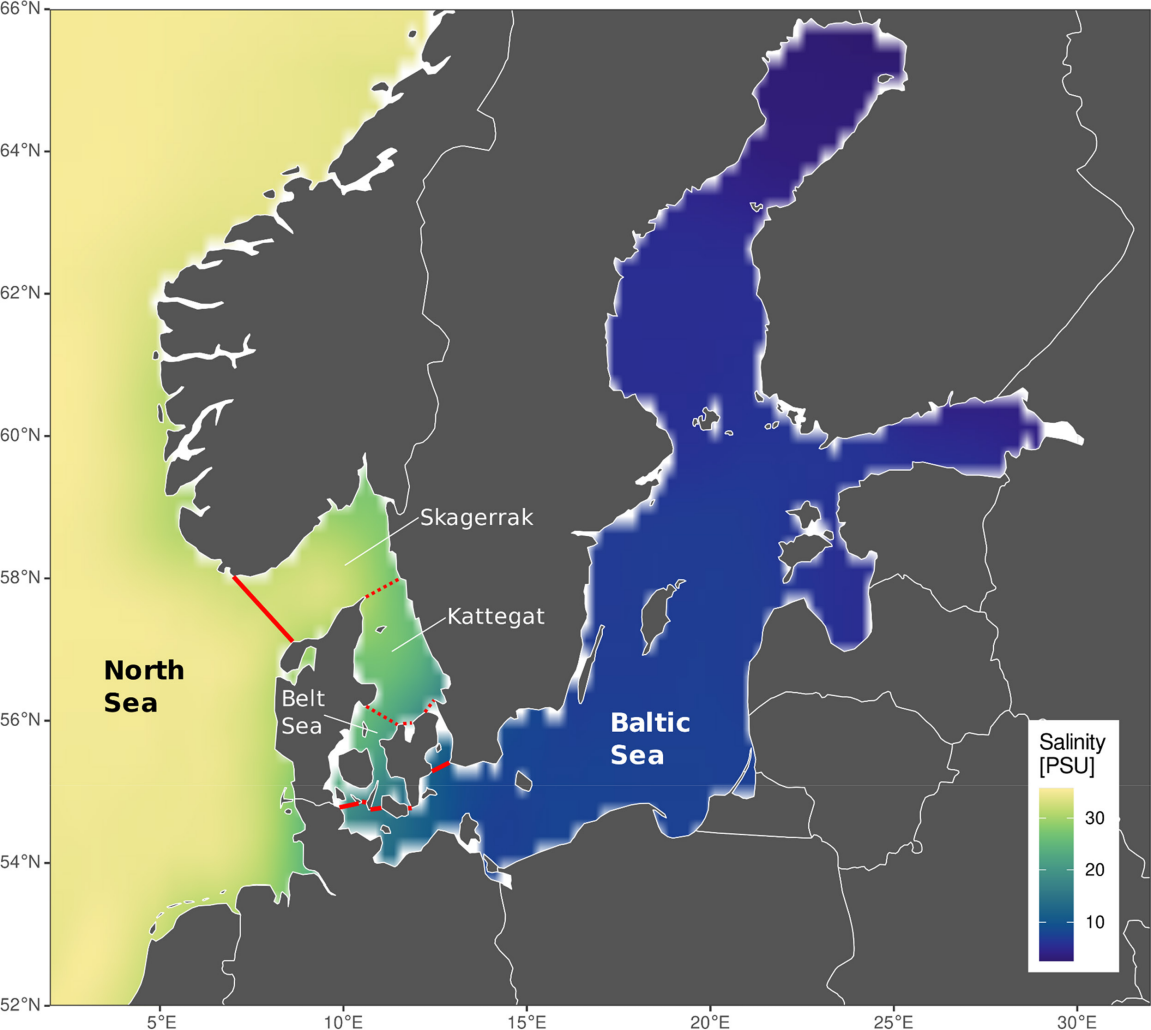
Map of the North Sea–Baltic Sea salinity gradient, showing the decadal interpolated average salinity from 2006 to 2015. Skagerrak, Kattegat, and Belt Sea comprise the transition zone between the North and Baltic Seas, that is, the area between the solid red lines. Salinity data from Hinrichs and Gouretski (2019) available at https://www.cen.uni‐hamburg.de/icdc/data/ocean/bnsc‐hyd.html

A marine region characterized by both restricted water movement and strong environmental gradients is the North Sea–Baltic Sea region. The Baltic Sea is the world's largest inland brackish water body with a west‐to‐east salinity gradient. The North Sea connects to the Baltic Sea via the narrow channels of Kattegat, Skagerrak, and the Belt Sea, which is littered with islands and bridges (Figure 1). Most marine organisms likely colonized the Baltic from the North Sea over the course of the past 8000 years, when the Baltic Sea turned from freshwater to brackish after the last glacial maximum (LGM), about 15,000 ya. During the preceding glaciation, the Baltic Sea was covered in ice. Before that, until about 200,000 ya, the geographic region of the Baltic Sea was no sea at all, but a landmass with a large river system (André et al., 2011). Numerous non‐native species from other regions of the world have colonized the Baltic Sea over the last centuries, either directly or indirectly introduced by humans. Examples are the Black Sea lineage of the shrimp *Palaemon elegans*, the crab *Hemigrapsus takanoi* from Japan and the clam *Mya arenaria* from North America (Behrends et al., 2005; Geburzi et al., 2015; Petersen et al., 1992; Reuschel et al., 2010). A few species, such as the blue mussel *Mytilus trossulus*, may have colonized the Baltic Sea from the White or Barents Sea during brief periods when it was connected to the Baltic Sea (Väinölä & Strelkov, 2011).

Despite the young evolutionary age of the Baltic Sea, at least two species evolved in the Baltic Sea, the brown algae *Fucus radicans* (Pereyra et al., 2009) and the Baltic flounder *Platichthys solemdali* (Momigliano et al., 2017). In both cases, adaptation to the salinity gradient has been invoked as the driving force of speciation. Adaptation may also play a role in ecological differentiation between populations in this habitat: In at least five fish species, North and Baltic Sea populations perform better in their natal salinity (Andersen et al., 2009; Berg et al., 2015; Gaggiotti et al., 2009; Guo et al., 2015; Larsen et al., 2008; Papakostas et al., 2012), as do the marine amphipods *Gammarus locusta* and *G*. *oceanicus* (den Hartog, 1964; Fenchel & Kolding, 1979; Segerstråle, 1947). Moreover, the jellyfish *Aurelia aurita* has differing reproductive cycles in different parts of the North and Baltic Sea system (Lucas, 2001). The marine diatom *Skeletonema marinoi* shows adaptive growth optima under North or Baltic Sea salinities, as well as genetic differentiation (Sjöqvist et al., 2015).

These findings may represent a general scheme for the North Sea–Baltic Sea transition zone as an ecologically driven barrier to gene flow. Corroborating evidence comes from a number of population genetic studies that found significant genetic differentiation across the North Sea–Baltic Sea environmental gradient for 23 species, including plants, crustaceans, priapulids, mollusks, mammals, and fish (Johannesson & André, 2006; Wennerström et al., 2013, 2017). Such general patterns of genetic differentiation are not only expected under ecological differentiation, but also consistent with neutral divergence processes caused by limited connectivity between the North and Baltic Sea. A few other species, such as the non‐native barnacle *Amphibalanus improvisus* and the mysid shrimp *Mysis mixta*, are not or only weakly genetically differentiated between North and Baltic Sea (Johannesson & André, 2006). These species do not necessarily contradict the idea of the North Sea–Baltic Sea transition zone as a barrier to gene flow. Instead, they may have colonized the Baltic Sea relatively late, such as the non‐native *A*. *improvisus*, not leaving enough time for observable genetic differences to arise. What constitutes “enough time” depends on a species’ demography: Large populations need longer to differentiate, as do species with a long generation time (Kingman, 1982). Even little gene flow, which we may expect based on intermittent saltwater inflow from the North Sea, slows down differentiation processes (Kimura & Maruyama, 1971).

It seems plausible that both ecological and neutral divergence processes occur, and the path each species takes depends on their life history and demography (Ewers‐Saucedo & Wares, 2020). Limited water exchange should influence species with a planktonic phase the most, while species with little dispersal ability might be fastest to adapt locally to environmental conditions (Kisdi, 2002; Schluter, 2000). Moreover, intrinsic environmental tolerances differ between species so that some species perceive an environmental barrier where others do not. This means that for some species, the entrance of the Baltic Sea may form a significant barrier to gene flow, while other species may cross into the Baltic unhindered.

We set out to test whether the Baltic Sea forms a significant barrier to gene flow, and whether this barrier may be due to limited connectivity or ecological adaptation. Limited connectivity should lead to significant genetic differentiation between North Sea and Baltic Sea populations at putatively neutral genetic loci, such as the mitochondrial cytochrome oxidase subunit 1 gene (COI). Ecological divergence can be inferred from basin‐specific salinity tolerances. Secondly, we identified life history traits that correlate with either evolutionary process. Given the diversity in life histories and population sizes, we focused this study on marine invertebrate species, and conducted population genetic simulations to understand the expected outcomes based on limited sampling and the evolutionary young age of the Baltic Sea.

## 2 METHODS

### 2.1 Acquisition of life history data

**FIGURE 2:**
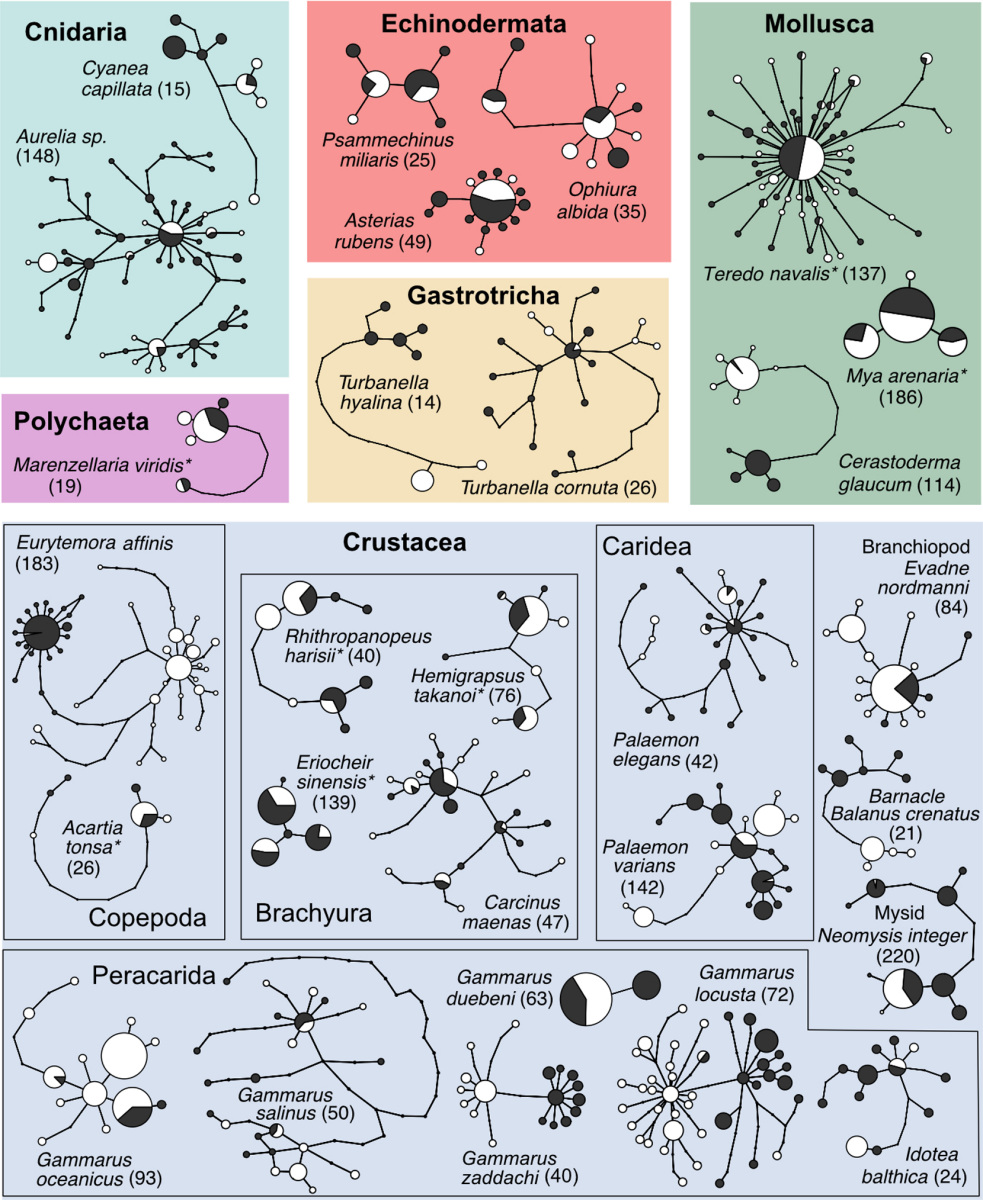
Haplotype networks for all 28 investigated species grouped by taxonomic affinity. Size of the circles is relative to the sample size, but not the same between species. Colors of the circles denote populations: black = North Sea, white = Baltic Sea, and asterisks denote non‐native species. Background colors distinguish between higher taxa. Overall sample size n in parentheses

Based on the availability of life history and genetic data, we identified a set of 28 species to be considered in this study, including members of the Cnidaria (2 spp.) Crustacea (17 spp.), Echinodermata (3 spp.), Gastrotricha (2 spp.), Mollusca (3 spp.), and Polychaeta (1 sp.) (Figure 2, Table 1). We retrieved information on pelagic larval duration (PLD), adult dispersal ability, adult habitat, and salinity tolerance for all investigated species from studies published throughout the 20th and 21st centuries. Our earliest literature sources dated back more than 100 years, and these historical studies may not always meet modern methodological standards, but were for several species the only available source of information. We included both experimental and observational estimates of salinity tolerance, and discriminated between estimates for populations from within and outside the Baltic Sea where possible. For little‐studied species and/or species that are difficult to rear/brood in the laboratory, we had to estimate PLD and salinity tolerance from studies not particularly addressing these traits. We decided to generally report salinity tolerance data for adult organisms, even though the larvae of many marine invertebrates are known to require higher salinities to undergo full development (see, e.g., Anger, 2001, for crustaceans; Sherman et al., 2016). However, larval salinity tolerances were available for very few of the investigated species only, and were lacking for Baltic Sea populations (with the exception of *Carcinus maenas*; see Results section). As a further proxy for salinity tolerance, we recorded the eastern most longitude at which a species was reported consistently in the Baltic Sea, based on the OBIS (Ocean Biodiversity Information System, https://obis.org) and GBIF (Global Biodiversity Information Facility, www.gbif.org) databases, as well as distribution records in the literature. When retrieving data from OBIS or GBIF, we excluded isolated data points and data points prior to 1990, because salinity may have changed, and we wanted a comparable picture of salinity tolerance. For non‐native species, we also searched for the year of their first records in the North and Baltic Seas, respectively.

**TABLE 1:** Life history traits of the investigated species. For non‐native species, the year of the first record for North and Baltic Seas is given. Salinity tolerances are derived from either experimental (exp) or observational (obs) studies. They are given for populations outside the Baltic Sea and, where available, Baltic Sea populations; bold numbers highlight differences in lower salinity tolerance limits inside and outside the Baltic Sea of ≥2 PSU. Easternmost longitudes are derived from the OBIS database, except for three species (see footnotes)

Species (Abbreviation)	Status/Introduction		Adult habitat	Adult range	Rafting/drifting	Development	Min. PLD (days)	Salinity tolerance (PSU)		Easternmost longitude
	North S.	Baltic S.						Outside Baltic S.	Inside Baltic S.	
**Cnidaria**										
*Aurelia* sp. (AuS)	Native		Pelagic	km	Yes	Planktonic	5	**13**–35^obs^	**5**–31^obs^	24.95
*Cyanea capillata* (CyC)	Native		Pelagic	km	Yes	Planktonic	4	**12**–36^exp^	≥**7** ^obs^	12.92
**Crustacea**										
*Acartia tonsa* (AcT)	1916	1925	Pelagic	m	Yes	Planktonic	19	1–72^exp^	2–33^exp^	27.25
*Balanus crenatus* (BaC)	Native		Benthic	sessile	Yes	Planktonic	14	**14**–55^exp^	≥**10** ^obs^	14.51
*Carcinus maenas* (CaM)	Native		Benthic	m	No	Planktonic	55	4–52^?^		13.50
*Eriocheir sinensis* (ErS)	1912	1926	Benthic	km	No	Planktonic	15	0.1–32^obs^		21.00
*Eurytemora affinis* (EuA)	Native		Pelagic	km	Yes	Planktonic	11	0.1–40^obs^	0.5–20^obs^	29.35
*Evadne nordmanni* (EvN)	Native		Pelagic	km	Yes	Brooded	–	1–35.4^obs^		29.35
*Gammarus duebeni* (GaD)	Native		Benthic	m	Yes	Brooded	–	0.1–48^obs^	1–85^obs^	27.83
*G*. *locusta* (GaL)	Native		Benthic	m	Yes	Brooded	–	**15**–33^obs^	≥**5.5** ^obs^	24.37
*G*. *oceanicus* (GaO)	Native		Benthic	m	Yes	Brooded	–	**18**–33^obs^	**2.5**–41^obs^	27.92
*G*. *salinus* (GaS)	Native		Benthic	m	Yes	Brooded	–	1–31^obs^	≥2.5^obs^	27.98
*G*. *zaddachi* (GaZ)	Native		Benthic	m	Yes	Brooded	–	0.5–27^exp^	≥1^obs^	27.92^a^
*Hemigrapsus takanoi* (HeT)	1999	2014	Benthic	m	Yes	Planktonic	22	7–35^obs^		11.48^b^
*Idotea balthica* (IdB)	Native		Benthic	m	Yes	Brooded	–	**6**–35^exp^	≥**3.5** ^obs^	27.29
*Neomysis integer* (NeI)	Native		Pelagic	km	No	Brooded	–	0.1–38^obs^		29.35
*Palaemon elegans* (PaE)^c^	Native		Benthic	m	No	Planktonic	22	5–35^obs^		11.19
*Palaemon varians* (PaV)	Native		Benthic	m	No	Planktonic	38	2–45^obs^	≥0.5^obs^	14.37
*Rhithropanopeus harrisii* (RiH)	1874	1936	Benthic	m	No	Planktonic	12	0.5–40^exp^		24.50
**Echinodermata**										
*Asterias rubens* (AsR)	Native		Benthic	m	No	Planktonic	49	**18**–36^obs^	≥**8** ^obs^	14.28
*Ophiura albida* (OpA)	Native		Benthic	m	No	Planktonic	18	**20**–35^obs^	**10**–27^obs^	15.30
*Psammechinus miliaris* (PsM)	Native		Benthic	m	No	Planktonic	20	**18**–35^exp^	≥**12** ^obs,^ ^d^	12.28
**Gastrotricha**										
*Turbanella cornuta* (TuC)	Native		Benthic	m	No	Brooded	–	3–30^obs^		19.26^e^
*T*. *hyalina* (TuH)	Native		Benthic	m	No	Brooded	–	**5**–60^exp^	≥**2** ^obs^	18.35^e^
**Mollusca**										
*Cerastoderma glaucum* (CeG)	Native		Benthic	sessile	No	Planktonic	21	4–60^obs^		26.66
*Mya arenaria* (MyA)	1240		Benthic	sessile	No	Planktonic	10	4–35^obs^	≥4^obs^	25.80
*Teredo navalis* (TeN)	1730	1835	Benthic	sessile	Yes	Planktonic	11	5–35^exp^		13.10
**Polychaeta**										
*Marenzelleria viridis* (MaV)	1979	2005	Benthic	sessile	No	Planktonic	25	0.5–45^exp^		28.01

^a^ Easternmost literature record at 28.41°E (Segerstråle, 1947).

^b^ GBIF database; Geburzi et al. (2015).

^c^ Only the Atlantic type is considered here (see text for details).

^d^ Minimum salinity tolerance derived from occurrence data in the OBIS database.

^e^ Kolicka et al. (2014).

The shrimp *Palaemon elegans* is a special case, as two genetically highly divergent lines occur in the Baltic Sea. One of them is also present in the North Sea and Atlantic (“Atlantic type”), and one was most likely introduced to the Baltic Sea from the Black Sea (“Black Sea type”) (Reuschel et al., 2010). It is unclear whether these lineages hybridize. Their genetic distance suggests that they are separate species. We only consider the Atlantic lineage here, as the non‐native Black Sea lineage does not (yet) occur in the North Sea (pers. comm. A. Böttcher). We therefore disregarded life history and distribution data from regions in the Baltic Sea where the Black Sea type occurs according to Reuschel et al. (2010).

A full bibliography of the life history data sources is given in Appendix 1.

### 2.2 COI sequencing

Over the past ten years, bachelor's, master's, and doctoral students at the Zoological Museum in Kiel have investigated population genetic differences between North and Baltic Sea populations for a number of marine invertebrates. Out of these, seven species were considered in this study: three echinoderms, three brachyuran crabs, one caridean shrimp, and one barnacle (Table S2). For each species, the students extracted DNA from a maximum of 20 specimens each from North Sea and Baltic Sea using commercial DNA extraction kits (Roth, Stratec Molecular) or the Chelex method (Walsh et al., 1991). Student‐ and species‐specific information on the respective extraction protocol, primers, and PCR settings are available in the Table S1. The accession numbers for these new sequence data on NCBI GenBank (www.ncbi.nlm.nih.gov) are available in the Table S2 (column “New GenBank Acc.”).

### 2.3 Acquisition of genetic data

We searched for publicly available cytochrome oxidase subunit 1 (COI) sequence data of species from the western or central Baltic Sea and the North Sea. We began by extracting data from the studies cited in Johannesson and André (2006). To find newer publicly available sequence data especially for the Baltic Sea, we searched for articles citing Johannesson and André (2006), searched NCBI GenBank for “Baltic Sea” and “cytochrome oxidase,” and searched Google Scholar for “Baltic Sea phylogeography marine” and “Baltic Sea marine population.” A good source for North Sea data was recent large‐scale barcoding efforts for Crustacea (Raupach et al., 2015), Mollusca (Barco et al., 2016), and Echinodermata (Laakmann et al., 2016). Sequence data for the transition zone (as described in Figure 1) were generally rare, and we did not include them in our overall analyses, but utilized them to assess the location of genetic breaks where appropriate.

In most studies, the barcoding marker located at the 5’ end of the COI gene was amplified (Folmer et al., 1994). A list of all sequence data sources is found in Appendix 1. We downloaded sequence or haplotype data from NCBI GenBank, supplements of publications, or the Barcoding of Life Database website (www.boldsystems.org). For accession numbers for these downloaded data from both NCBI GenBank and the Barcoding of Life Database, see Table S2. When the sequence data represented haplotypes, rather than sequences for each sampled individual, we reconstructed haplotype frequencies from information within the respective publication.

We excluded data from several studies that sequenced different mitochondrial fragments or that did not provide enough information to reconstruct haplotype frequencies. In the case of the shipworm *Teredo navalis*, we included three locations that were sampled after 2012 and were not close to each other: Kiel, Kühlungsborn, and Hiddensee, to reduce the otherwise very large number of sequences. In accordance with the life history data, we only used COI data from the Atlantic lineage of *Palaemon elegans* (see above).

### 2.4 Data quality control

We excluded species that had not colonized the Baltic Sea directly via the North Sea or vice versa. However, we kept species where the colonization may have proceeded from the Baltic Sea to the North Sea, as in some brackish water species. We removed highly divergent sequences from cryptic or misidentified species. For each species, we aligned all COI sequences in Geneious v.9.1.8 (Kearse et al., 2012) with the “Map to reference” function, using the longest sequence as reference. This appeared to be a faster, more reliable alignment approach than aligning with a “Multiple align” algorithm. We checked the alignment for gaps, removed short sequences, and trimmed all remaining sequences to the same length. This means that different species have different alignment lengths. We removed species with a final alignment length below 400 bp, as shorter sequences are likely to harbor less genetic diversity, and thus may lead to underestimates of diversity and differentiation.

### 2.5 Population genetic analyses

All analyses were conducted in the R environment (R Core Team, 2019). For each species, we reconstructed haplotype networks using the “haplotype” function of the package “haplotypes” (Aktas, 2015). We calculated haplotype diversity of each population (Nei & Tajima, 1981) with the function “hap.div,” and nucleotide diversity (Nei, 1987) with the function “nuc.div,” both available in the “pegas” package (Paradis, 2010). We tested for significant differences in the genetic diversity of the North and Baltic Sea by conducting analyses of variance (ANOVA) for each species and diversity measure using custom scripts available online (https://doi.org/10.6084/m9.figshare.c.5341910). We calculated Tajima's *D* and its deviation from zero with the function “tajima.test” of the “pegas” package (Paradis, 2010). Tajima's *D* is the test statistic that calculates the difference between the expected genetic diversity based on the number of segregating sites and the average number of pairwise differences (Tajima, 1989). A negative Tajima's *D* indicates either a recent selective sweep or population expansion after a bottleneck, the expectation for relatively recent colonization.

We calculated genetic differentiation between North and Baltic Sea populations as *Φ*_ST_ with the function “pairwiseTest” of the package “strataG” (Archer et al., 2017) and Jost's *D* with the function “pairwise_D” of the “mmod” package (Winter, 2012), and wrote our own function to calculate the nearest neighbor statistic Snn (Hudson, 2000). *Φ*_ST_ is a derivative of the classical fixation index F_ST_, adapted for mitochondrial haplotype data (Excoffier et al., 1992). Jost's *D* is supposed to be a more accurate measure of population differentiation when genetic diversity is high and the number of unique alleles per population is large (Jost, 2008). Snn is particularly powerful when sample sizes are small or uneven between populations (Hudson, 2000). We estimated significant deviations from zero (no differentiation between population pairs) for all differentiation indices by comparing the point estimates with an empirical distribution of values based on 1000 permutations.

### 2.6 Rarefaction analysis

Initially, we included species for which at least five sequences for each population were available. This low number is sufficient to distinguish between high‐ and low‐diversity populations (Goodall‐Copestake et al., 2012). To ensure that any observed lack of genetic differentiation was not due to small sample size (i.e., lack of power), we randomly subsampled all species in which populations had more than 20 sequences to 5, 10, or 15 sequences per population, and recalculated population genetic estimates on these random subsamples. We repeated the subsampling 100 times and compared the distribution of these estimates with the point estimates of the full dataset for each species. We also repeated all population genetic analyses on datasets rarefied to the same number of sequences per population. In this last iteration, different species can have different sample sizes, but the sample sizes are the same for each population within a species.

### 2.7 Coalescent estimates of theta and migration rate

Differentiation indices such as *Φ*_ST_ assume, among others, that migration rates are symmetric. The coalescent approach, on the contrary, allows migration rates to vary (Beerli, 2006), which is relevant for testing the hypothesis of the Baltic Sea as a population sink. For all species with more than 20 sequences per population, significant population differentiation indices, and clearly separated populations based on the haplotype networks, we estimated the mutation‐rate scaled migration rates between North and Baltic Sea populations m, the mutation‐rate scaled effective population size q of each population, and the time since divergence t, implemented in the software “IMa2” v.8.27.12 (Hey & Nielsen, 2004). IMa2 uses Bayesian inference to estimate posterior probability densities of these population genetic parameters. It is particularly well suited for populations that diverged recently (Hey & Nielsen, 2004). We used the HKA model of sequence evolution, exponential priors for m, and uniform priors for q and t, with species‐specific upper bounds for *q*. We conducted several short preliminary runs for each species to determine priors that capture the full range of posterior probabilities. We started by choosing an upper prior for theta that was five times our estimate of nucleotide diversity multiplied by sequence length, as suggested in the IMa2 manual. We increased the upper prior when the output of the preliminary run indicated that the posterior probabilities were strongly right‐skewed, and decreased the upper prior when the posterior probabilities were strongly left‐skewed toward zero. These preliminary runs showed that a splitting time t of 10 was appropriate for all species, which increases comparability between them. For the final run, the burn‐in period was 10,000 steps, and the record period 1,000,000 steps, with the results saved every 100 steps for a total of 10,000 genealogies. We employed the low heating scheme with 20 chains (‐hfg ‐hn20 ‐ha0.96 ‐hb0.9) described in the IMa2 manual, and replicated each run three times to confirm convergence. For details on each species, see the output files that are available online (https://doi.org/10.6084/m9.figshare.c.5326685). Convergence was further ensured by high effective sampling sizes (ESS), single posterior probability density peaks, and zero posterior probabilities at the upper limit of the distribution. Divergence times were converted to years by dividing them by the mutation rate per year scaled to the respective alignment length. We based this mutation rate on a substitution rate of 1.22% per one million years, which appears to be similar across marine invertebrates (Wilke et al., 2009).

### 2.8 Correlations between genetic and life history data

The investigated non‐native species have been present in the North and Baltic Sea for at most 780 years (the soft shell clam *Mya arenaria*). Thus, we do not expect them to “follow the same rules” as native species, who evolved in the North Sea–Baltic Sea system. We therefore excluded non‐native species from the following analyses. We estimated the effects of life history on either genetic differentiation between North and Baltic Sea or salinity tolerance, which we approximated by the easternmost longitude a species was recorded from in the Baltic Sea, as well as the effects of these two factors on each other. We used the Bayesian approach implemented in the “MCMCglmm” package using the “mcmcglmm” function (Hadfield, 2010). All life history traits were treated as fixed effects to assess their significance. In particular, we included the dispersal potential of larvae, the dispersal potential of adults, and the taxon in the models. Given that these models are overparameterized for our sample size and the number of species, we sequentially removed response variables that were nonsignificant from the model. We used the default priors and let the model run for 60,000 generations. Significance was assessed using the mcmc p‐value (pMCMC). To ensure convergence, we inspected the traces and checked the posterior densities visually for normality, and made sure the effective sample size was larger than 200 in all variables.

## 3 RESULTS

### 3.1 Acquisition of life history data

The majority of species is benthic (22), their adult mobility typically being in the range of meters. Five species (*Balanus crenatus*, *Cerastoderma glaucum*, *Marenzelleria viridis*, *Mya arenaria*, and *Teredo navalis*) are sessile as adults. A planktonic larval phase occurs in 18 of the investigated species, four of them being pelagic and 14 being benthic as adults, including all sessile species. Pelagic larval duration (PLD) ranges from less than a week in the cnidarians *Aurelia* sp. and *Cyanea capillata* to six weeks and more in *Asterias rubens* and *Carcinus maenas* (Table 1). The investigated species thus cover a broad range of dispersal potentials when jointly considering adult mobility and the presence and duration of a planktonic larval phase.

At the upper extreme are the two cnidarian species, with highly mobile, pelagic adults and planktonic larvae. Among the benthic species, *E*. *sinensis* stands out with highly mobile adults, capable of long‐distance migrations, and a PLD of about two weeks. Ten further benthic species have a PLD exceeding two weeks. In all *Gammarus* species, as well as *Idotea balthica*, long‐range dispersal of adults may occur by individuals rafting on floating macroalgae or seagrass, potentially “boosting” adult mobility under favorable conditions. At the lower extreme are the two gastrotrich species that live in‐benthic as adults, attach their eggs to sand grains within the sediment, and lack a planktonic larval phase.

**FIGURE 3:**
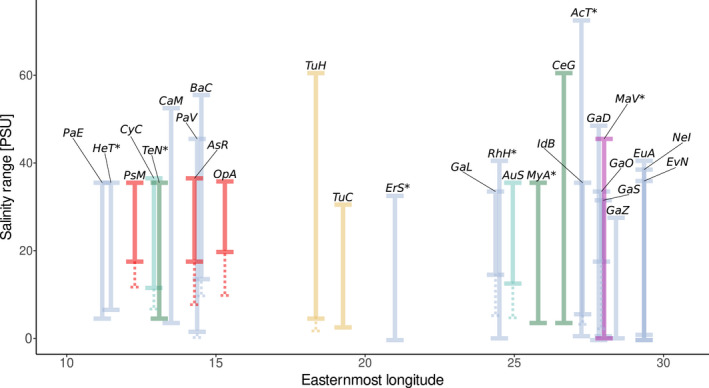
Salinity tolerances and eastern distribution limit in the Baltic Sea of the investigated species. Dotted lines indicate enhanced low‐salinity tolerance in Baltic Sea populations in species where Baltic Sea‐specific salinity tolerance data were available. Bar colors indicate the higher taxonomic group of each species (compare Figure 2), and asterisks denote non‐native species. For species abbreviations, see Table 1

Most of the investigated species have broad‐to‐very broad salinity tolerances, ranging from (nearly) freshwater to fully marine or even hypersaline conditions. In general, the further east a species occurs into the Baltic Sea (i.e., the more brackish conditions it tolerates), the broader its salinity range appears to be (Figure 3). For *Aurelia* sp., *C*. *capillata*, *Balanus crenatus*, *Gammarus locusta*, *G*. *oceanicus*, *Idotea balthica*, *Palaemon varians*, *Asterias rubens*, *Ophiura albida*, *Psammechinus miliaris*, and *Turbanella hyalina*, we found explicit literature evidence that their Baltic Sea populations tolerate lower salinities compared with North Sea/Atlantic populations (Table 1). This difference was most pronounced in *G*. *oceanicus*, *A*. *rubens*, and *O*. *albida*, with their Baltic Sea populations tolerating water less saline by 10 PSU and more compared to their North Sea populations. *Carcinus maenas* is a special case when comparing salinity tolerances, as adults from North and Baltic Sea populations do not seem to differ in their salinity tolerances, but the Baltic Sea populations of *C*. *maenas* are able to complete larval development at much lower salinities compared to their North Sea conspecifics (13 vs. 20 PSU; Anger et al., 1998; Dries & Adelung, 1982).

Seven of the investigated species are considered non‐native in the Baltic Sea: *Acartia tonsa*, *Eriocheir sinensis*, *Hemigrapsus takanoi*, *Rhithropanopeus harrisii*, *Mya arenaria*, *Teredo navalis*, and *Marenzelleria viridis*. Most non‐native species have a long planktonic larval phase (PLD > 2 weeks, except *R*. *harrisii* and *M*. *arenaria*; Table 1), a typical trait of successful invasive marine invertebrates. Time since introduction in the Baltic Sea varies between almost 800 years (*Mya arenaria*; Behrends et al., 2005; Petersen et al., 1992) and eight years (*Hemigrapsus takanoi*; Geburzi et al., 2015). The easternmost longitude of regular occurrence in the Baltic Sea corresponds fairly well with the salinity tolerance for most of the non‐native species. Only the recently established *H*. *takanoi* has its current eastern distribution limit distinctly west of the 7 PSU isohaline (compare Figures 1 and 3).

### 3.2 Acquisition of genetic data

Population sample sizes for the 28 species we considered in our study ranged from 14 to 220 sequences with a median of 25 sequences, and alignment lengths varied from 423 to 675 bp with a mean of 545 bp. For eight species, sequences were available for the transition zone as defined in Figure 1. Of these species, three were highly differentiated: *Cerastoderma glaucum*, *Eurytemora affinis*, and *Balanus crenatus*. This allowed us to clearly assign the transition zone sequences to either the North or Baltic Sea population (see Figure S1.1). From this, we inferred the break between North and Baltic Sea populations: between the Limfjord and North Sea (*C*. *glaucum*), between Skagerrak and Kattegat (*B*. *crenatus*), and between Skagerrak and the North Sea (*E*. *affinis*). In the case of *E*. *affinis*, we also included the few available White Sea sequences, which clustered with the North Sea sequences.

### 3.3 Rarefaction analysis

**FIGURE 4:**
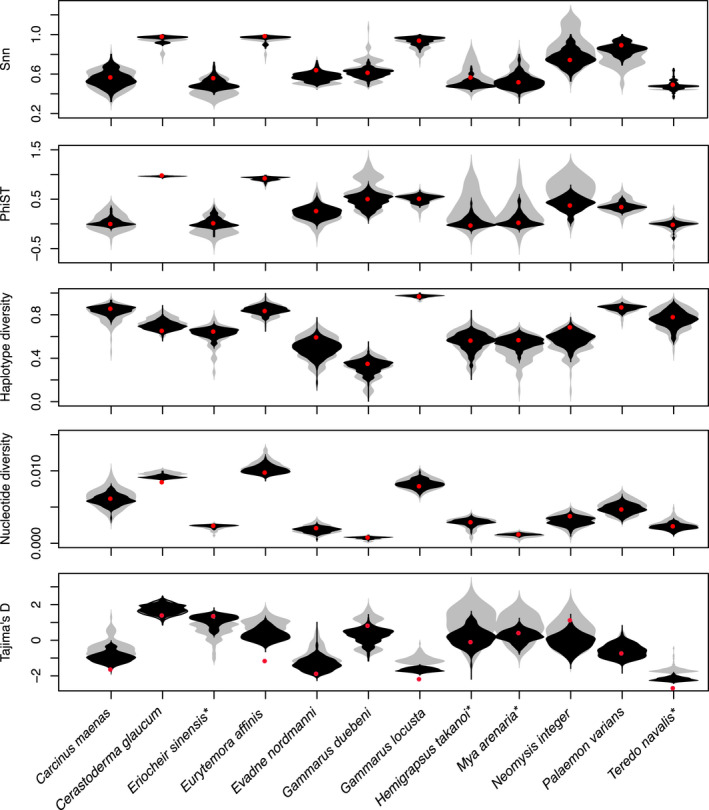
Population genetic rarefaction results for all species with more than 20 sequences per population. Red dots represent the point estimate based on the full dataset; gray violin plots, the distribution of estimates based on five random sequences per population; and black violin plots the distribution of estimates based on 10 random sequences. Asterisks denote non‐native species

Calculating genetic differentiation from only five sequences per population for all 10 species with more than 20 available sequences per population generated large ranges of differentiation index values. For *Φ*_ST_, the 95% range of estimated values was 0.83 averaged across all 10 species, and for Snn, this interval was 0.36 (Figure 4). Given that *Φ*_ST_ ranges from 0 to 1 and Snn from 0.5 to 1, these ranges are comparably wide. While the range was wide, the majority of estimates centered around the value calculated from the full dataset. In some species, we observed an upward bias, such that *Φ*_ST_ and Snn would be larger when sample sizes are small. The range for species with very high genetic differentiation (*Cerastoderma glaucum*, *Eurytemora affinis*, and *Gammarus locusta*) was much lower, suggesting that for highly differentiated species, small sample sizes provide accurate results. Increasing the sample size reduced the 95% intervals of estimated *Φ*_ST_ and Snn values (see Figure 2); variability of *Φ*_ST_ values reduced more with larger sample sizes than variability of Snn values.

Measures of genetic diversity were relatively robust to small sample size with relatively small ranges (Figure 4). Nucleotide diversity appeared to be a robust measure of genetic diversity. In contrast, Tajima's *D* had a wide range of values even at larger sample sizes (Figure 4). In some instances, the ranges did not even include the value estimated from the full dataset. Thus, this test statistic is only useful when sample sizes are large in comparison with the number of haplotypes. Considering that Tajima's *D* is based on the abundance of rare alleles, this result is expected. As a result of these rarefaction analyses, *Φ*_ST_ will be considered as the most robust measure of differentiation in the subsequent analyses, and nucleotide diversity the most robust point estimate of diversity. Tajima's D, on the contrary, will be considered less valuable for species with small sample sizes. We did not remove such species as they may add to our understanding of the role of the North Sea–Baltic Sea transition zone as an ecological–genetic barrier, especially when they are strongly differentiated.

### 3.4 Population genetic analyses

**FIGURE 5:**
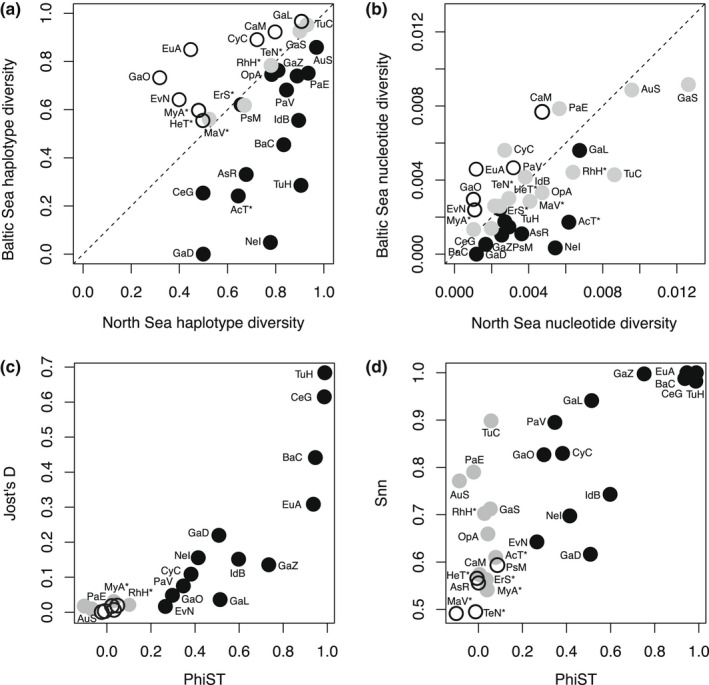
Population genetic comparison of North and Baltic Sea populations of marine invertebrates. (a) Haplotype diversity and (b) nucleotide diversity. Black: more diverse in North Sea, white: more diverse in Baltic Sea, and gray: equally diverse. (c) Comparison between the differentiation indices *Φ*_ST_ and Jost's *D*, (d) comparison between the differentiation indices *Φ*_ST_ and Hudson's Snn. Black: significantly differentiated with both indices, gray: only differentiated with Jost's *D* (c) or Hudson's Snn (d), and white: undifferentiated with either index. For species abbreviations, see Table 1

Genetic diversity varied considerably between species and populations. Only five species had even haplotype diversities in the North and Baltic Sea, whereas eight species had higher haplotype diversity in the Baltic Sea, and 13 species had lower haplotype diversity in the Baltic Sea (Figure 5a). The Baltic Sea population of the amphipod *Gammarus duebeni* had a haplotype diversity of 0, which means only a single haplotype was found in the Baltic Sea. This species also had only two haplotypes in the North Sea, making its genetic diversity extremely low (compare Figure 2). No North Sea population, on the contrary, had a haplotype diversity lower than 0.4. With regard to nucleotide diversity, 14 species were equally diverse in the North and Baltic Sea, and five species were more diverse in the Baltic Sea than in the North Sea (Figure 5b). The remaining eight species were more diverse in the North Sea. The ratio of nucleotide diversities from North and Baltic Sea populations was strongly correlated with the respective ratio of haplotype diversities (Pearson's product–moment correlation = 0.722, 95% confidence interval = 0.472, 0.865, *p*‐value <.001). Only the amphipod *Gammarus locusta* had a significantly higher haplotype diversity in the Baltic Sea than in the North Sea, but the nucleotide diversity showed the opposite trend.

The two differentiation indices *Φ*_ST_ and Jost's *D* gave qualitatively similar results (Figure 5c). *Φ*_ST_ identified about half of the species as significantly differentiated. Jost's *D* identified three additional species, the shrimp *Palaemon elegans*, and the two non‐native species with the oldest introduction date, the soft shell clam *Mya arenaria* and the crab *Rhithropanopeus harrisii*, as significantly differentiated. Hudson's Snn identified five additional species as significantly differentiated for a total of 22 species (Figure 5d). Five species were considered undifferentiated by all test statistics, three non‐native species (Figure 5c,d) and two native echinoderms, the sea star *A*. *rubens* and the sea urchin *Psammechinus miliaris*.

For 17 species, Tajima's *D* was not significantly different from zero for either North Sea or Baltic Sea population (see Figure S1.3). Four species (*Gammarus locusta*, *Turbanella hyalina*, *Acartia tonsa*, and *Neomysis integer*) had significantly negative values for the Baltic Sea population, while *Palaemon varians* had a significantly positive value in the Baltic Sea. *Evadne nordmanni* had a significantly negative value in the North Sea. Two species had Tajima's *D* values that were smaller than zero in both populations: *Teredo navalis* and *Eurytemora affinis*. The large number of insignificant values matches our rarefaction analysis, which showed that only large sample sizes estimate Tajima's *D* with confidence.

### 3.5 Coalescent estimates of theta and migration rate

For 13 of the 28 investigated species, more than 20 sequences were available for each population (Table S2). Of those, four species were significantly differentiated between the North and Baltic Sea populations based on the differentiation indices and a visual inspection of the haplotype networks: the cockle *Cerastoderma glaucum*, the amphipod *Gammarus locusta*, the shrimp *Palaemon varians*, and the copepod *Eurytemora affinis*.

**FIGURE 6:**
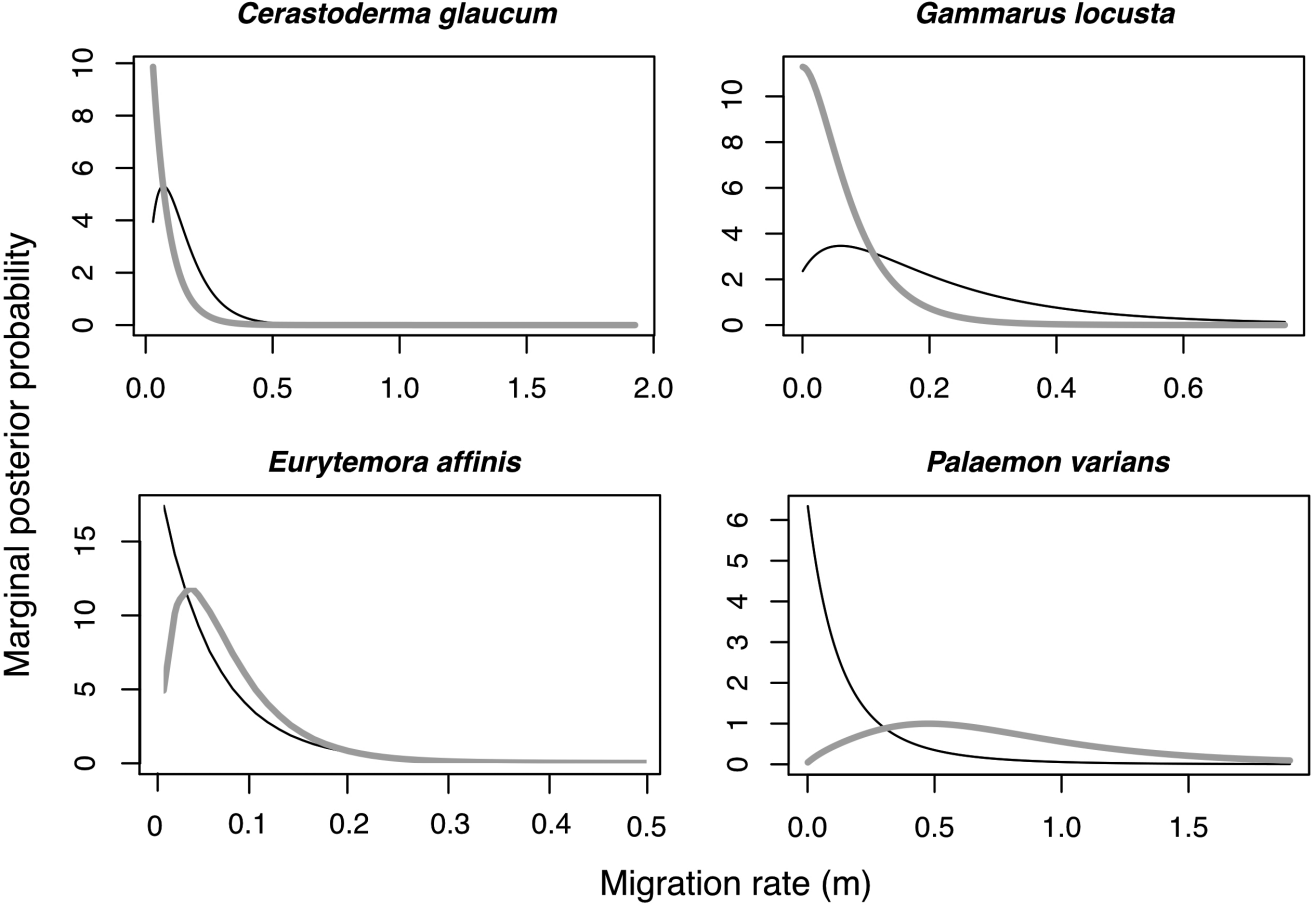
Distribution of posterior probabilities for mutation‐rate scaled population size q for species with more than 20 sequences per population and distinct population differentiation. The maximal values displayed for q denote the upper bounds of the uniform prior. The distribution of the Baltic Sea population is shown as a gray line; the distribution of the North Sea population, as a black line; and the distribution of the ancestral population, as a dashed line

**FIGURE 7:**
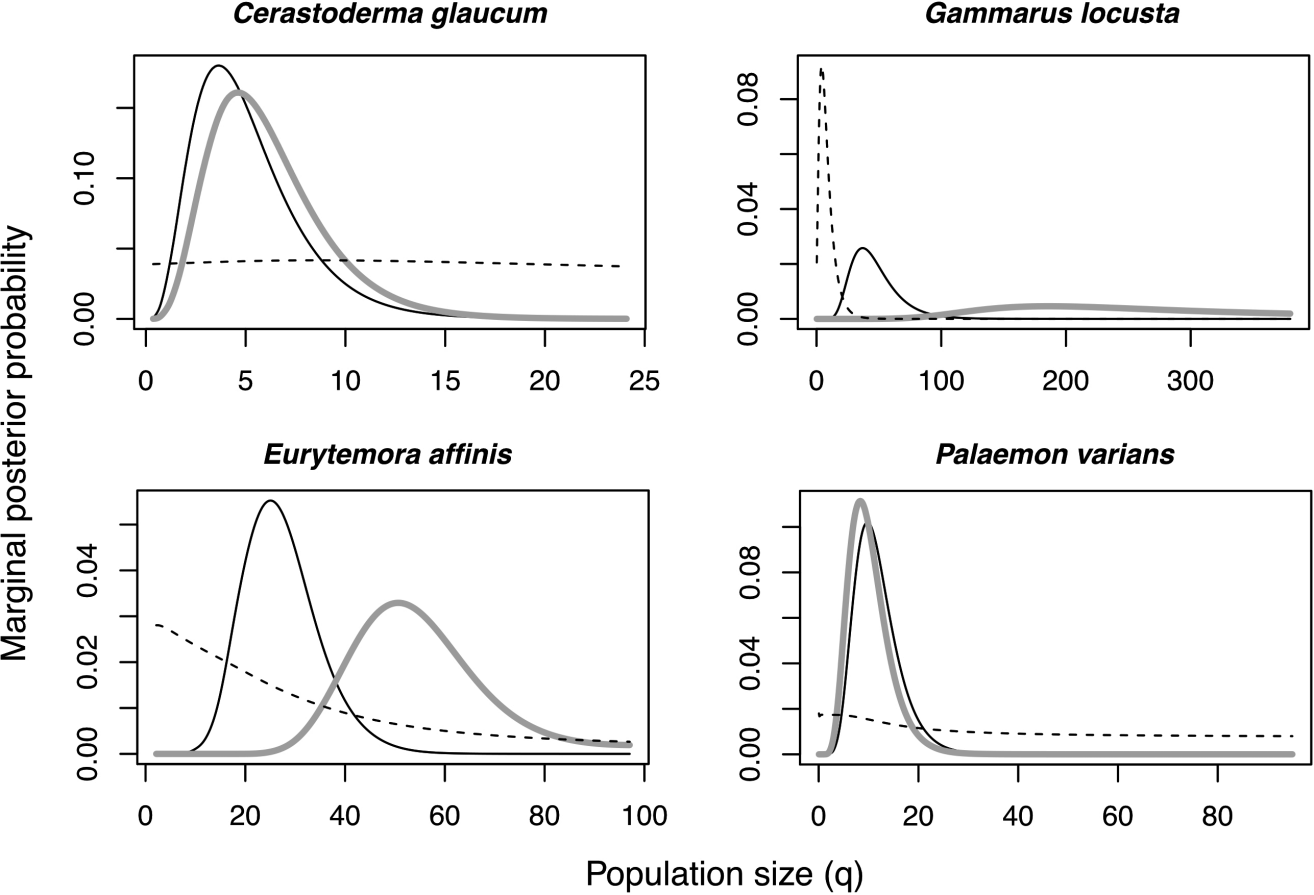
Distribution of posterior probabilities for mutation‐rate scaled population size q for species with more than 20 sequences per population and distinct population differentiation. The maximal values displayed for q denote the upper bounds of the uniform prior. The distribution of the Baltic Sea population is shown as a gray line, and the distribution of the North Sea population as a black line

These species converged on specific estimates with single peaks in the marginal posterior probability, zero probability at the upper bound, and ESS larger than 1300 for all estimates. The main issue with the analysis was that for each species, some estimates had very broad peaks (see Figures 6 and 7). These broad distributions may be attributable to a scarcity of data in relation to the effective population size. For the cockle *C*. *glaucum*, the copepod *E*. *affinis*, and the amphipod *G*. *locusta*, population sizes were larger in the Baltic Sea than in the North Sea, whereas the opposite was the case for the shrimp *P*. *varians* (Figure 6). Migration rates were larger from the Baltic Sea to the North Sea in the cockle *C*. *glaucum and* the amphipod *G*. *locusta*, and larger from the North Sea to the Baltic Sea for the copepod *E*. *affinis* and the shrimp *P*. *varians* (Figure 7).

IMa2 also estimated divergence times between populations. The four species had wide 95% HPD intervals: 98,004–1,570,498 years (*Palaemon varians*), 402,384–901,640 years (*G*. *locusta*), 232,456–1,492,743 years (*E*. *affinis*), and 146,601–1,581,838 years (*C*. *glaucum*) when assuming a generation time of one year for *P. varians* and *C. glaucum*, and a generation time of ½ year for the copepod *E. affinis* and the amphipod *G. locusta*. These confidence intervals overlap roughly between 400,000 and 900,000 years ago, indicating a possible concurrent colonization event at that time.

### 3.6 Correlations between genetic differentiation, salinity tolerance, and life history data

**FIGURE 8:**
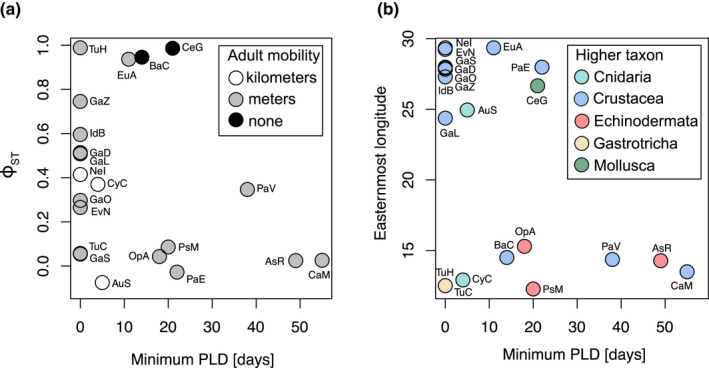
Correlations of pelagic larval duration (PLD) with ecological and genetic variables. (a) Genetic differentiation vs PLD, colors indicate adult mobility. (b) Easternmost longitude (which correlates strongly with salinity; see Figure 1) vs PLD, colors indicate higher taxonomic affinity

Life history and dispersal traits did not have significant linear relationships with the level of genetic differentiation. The MCMCglmm results showed that *Φ*_ST_ itself was significantly larger than zero across all native species, indicating significant differentiation across the North and Baltic Sea (pMCMC = 0.000335). Conversely, higher taxonomic units, adult mobility, or minimum PLD did not significantly correlate with *Φ*_ST_ (Figure 8a). However, the species with the longest PLD were little differentiated (*Carcinus maenas*, *Asterias rubens*) (Figure 8a). Genetic differentiation varied greatly between species without planktonic larvae. For species with planktonic larvae, genetic differentiation was either absent or very pronounced (Figure 8a). It does stand out that none of the investigated Echinodermata and almost none of the Mollusca were significantly differentiated between the North and Baltic Sea. All investigated alien species have low levels of population differentiation (Figure 5), which is expected given their relatively recent introduction into the Baltic Sea.

The easternmost longitude at which a species was reported in the Baltic Sea is a proxy of its natural salinity tolerance, as salinity declines to the east (Figure 1). This easternmost longitude was significantly and negatively affected by the minimum PLD (pMCMC = 0.01122) and the taxonomic affinity (Figure 8b). In particular, Mollusca were found further to the east (pMCMC = 0.00536), although this result is based on a single species, the cockle *Cerastoderma glaucum*.

## 4 DISCUSSION

**FIGURE 9:**
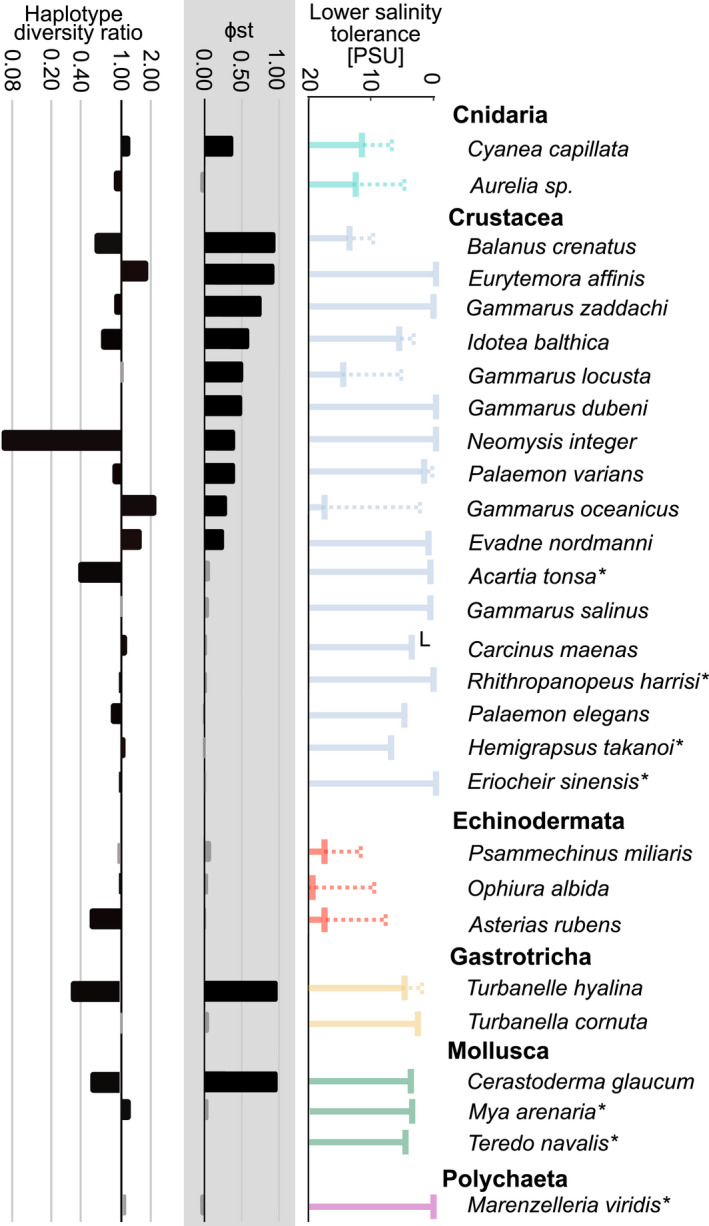
Ecological–genetic summary relevant to assess the potential of the North Sea–Baltic Sea transition zone as an ecological–genetic barrier. Left: nucleotide diversity ratio between Baltic Sea and North Sea populations; black bars indicate significant differences between Baltic and North Sea nucleotide diversity. Center: genetic differentiation (*Φ*_ST_) between Baltic Sea and North Sea populations; black bars indicate significant differentiation. Right: salinity tolerance; dotted bars indicate a lower salinity tolerance limit in Baltic Sea populations; a superscripted “L” indicates that this was only shown for the larvae. Colors indicate higher taxonomic groups (compare Figure 2), and asterisks indicate non‐native species

In this comparative study, we compiled genetic, ecological, and life history data for 28 marine invertebrate species that occur in both North and Baltic Sea. These species live under the marine conditions of the North Sea and under the brackish conditions of the Baltic Sea. We asked whether these species perceive the transition from the marine North Sea to the brackish Baltic Sea as a genetic and ecological barrier. Taking all of the available evidence together, we identified significant ecological and/or genetic differentiation for 18 of the 28 investigated species (Figure 9). For these 18 species, the entrance to the Baltic Sea represents a barrier to gene flow. Ten species were not genetically differentiated, seven of which are non‐natives (Figure 9). For these seven non‐natives, the lack of population differentiation and often equal genetic diversity between North and Baltic Sea are the result of their recent expansion into the Baltic Sea rather than long‐term connectivity between both basins. Moreover, for the majority of non‐native species, no data on ecological adaptation to the lower salinity of the Baltic Sea exist. The native gastrotrich *Turbanella cornuta*, which does not show signs of genetic differentiation, has also not been assessed ecologically. The shrimp *Palaemon elegans* is significantly differentiated using two of the three differentiation indices, and the amphipod *Gammarus salinus* is differentiated based on Hudson's Snn. Thus, for at least 85% of the native species, the transition between North Sea and Baltic Sea marks a genetic and ecological breakpoint, irrespective of their dispersal potential. Investigating the non‐native species may provide clues as to the timing of ecological adaptation, as would probing the genomes of native species for molecular signatures of adaptive evolution.

### 4.1 Genetic differentiation and gene flow

The three differentiation indices identified varying numbers of native species’ populations as significantly differentiated between the North and Baltic Sea: *Φ*_ST_ was the most conservative index, differentiating 62% of native species, Jost's *D* differentiated three additional natives, while Hudson's Snn was significant for over 90%. The four native and three non‐native species that were only identified by Snn had relatively small sample sizes, with the smallest population sample size per species ranging from six to nineteen, which may have caused insignificant *Φ*_ST_ values for some of these species. Our rarefaction analyses, however, suggested the opposite; species with small sample sizes have upward biased estimates. Alternatively, Snn overestimates genetic differentiation. Whatever the cause, these species appear not to be strongly differentiated between their respective North and Baltic Sea populations (Figure 4), but may be beginning to diverge. Curious are two of the oldest species introductions, the soft shell clam *Mya arenaria* (ca. 1240, Petersen et al., 1992) and the Harris mud crab *Rhithropanopeus harrisii* (ca. 1870, Wolff, 2005), which had significant Jost's *D* estimates. These species may be beginning to differentiate, which would make them ideal test cases to assess the speed of genetic and ecological differentiation. Given the evolutionarily young age of the Baltic Sea, the widespread genetic differentiation between North and Baltic Sea may be surprising. However, 8000 years translate to several thousand generations for marine invertebrates, and this is more than sufficient for drift to differentiate populations, especially if those populations are small (Barton et al., 2007). Our results confirm that gene flow between the North and Baltic Sea is generally limited. The same trend is apparent for seagrass, algae, fishes, and harbor seals (Johannesson & André, 2006; Wennerström et al., 2013), suggesting ubiquitous resistance to North Sea–Baltic Sea gene flow.

The connecting water body, the Belt Sea, is littered with islands, and in more recent times, bridges are restricting water movement. Continuous salinity measurements show that marine water inflow from the North Sea is restricted to the cold months in most years (Ewers‐Saucedo et al., 2020; Lennartz et al., 2014), which means that marine larvae, which predominantly disperse in spring and summer, will not be transported into the Baltic Sea. Our results support this limited connectivity, although it should not matter for species with highly motile adults, such as jellyfish and pelagic copepods. These species are nonetheless differentiated, which either means that very few North Sea individuals reach the Baltic Sea or that these do not survive well under Baltic Sea conditions. Models of oceanographic connectivity for the transition zone show that locations in the Skagerrak and Kattegat are connected and migration occurs mostly from the Kattegat to the Skagerrak (Godhe et al., 2013), but that oceanographic connectivity dropped significantly when entering the Baltic Sea (Sjöqvist et al., 2015).

Support for the adaptive hypothesis comes from three highly differentiated species (*Cerastoderma glaucum*, *Eurytemora affinis*, and *Balanus crenatus*), for which sequence data from the transition zone between North and Baltic Sea exist. If limited dispersal is responsible for the observed pattern, the phylogeographic break between differentiated lineages should most likely be the Belt Sea. However, the Baltic Sea lineage of all three species extended past the Belt Sea, with breaks as far west as the Skagerrak. If the mitochondrial data are congruent with the rest of the genome, this points to an ecological maintenance of the two lineages, rather than a purely neutral divergence. In general, a more detailed genetic and ecological analysis of the transition zone would be highly informative.

### 4.2 Non‐native species and human‐mediated gene flow

The differentiation index *Φ*_ST_ was nonsignificant for the seven investigated non‐native species. Superficially, this result seems to counter the argument of limited connectivity between the North and Baltic Sea, as the lack of differentiation could indicate unhindered natural dispersal into the Baltic Sea. However, we consider human‐mediated dispersal to play a crucial role in the colonization and differentiation processes of non‐native species in the Baltic Sea. Non‐native species are predisposed for anthropogenic dispersal, as this is the reason they are non‐native in the first place. The life history of native, noninvasive species may make transport with ships into the Baltic Sea less likely. Furthermore, differential local adaptation of populations from different parts of their native range may lead to interspecific priority effects that prevent establishment and admixture of native populations, even if they are transferred by human activities (Makino et al., 2018). An exception is the shore crab *Carcinus maenas*, which is invasive in many parts of the world (Carlton & Cohen, 2003). Comparable to the non‐native species in our study, it has no genetic differentiation between North and Baltic Sea. Thus, for this species, we may also assume repeated transport between both basins. Ship traffic, particularly via the Kiel Canal, has been identified as the most likely introduction pathway for the crabs *R*. *harrisii* and *H*. *takanoi* (Nehring, 2000; Geburzi et al., 2015). It is generally considered one of the most important invasion vectors to the Baltic Sea (Leppäkoski et al., 2002; Ojaveer et al., 2017). Although not being a vector *sensu stricto*, the Kiel Canal itself provides an anthropogenic invasion corridor for species capable of long‐distance migration such as the Chinese mitten crab *Eriocheir sinensis*. While natural dispersal of *E*. *sinensis* around the Danish peninsula into the Baltic Sea would have likely taken several decades considering the dating of records from Danish coasts, it had successfully crossed the Kiel Canal west to east only six years after its first occurrence on the German North Sea coast (Herborg et al., 2003). In general, the high rate of ship traffic between the North and Baltic Seas may well cause repeated/continued introductions of non‐native species that prevent differentiation of introduced Baltic Sea populations (compare Roman & Darling, 2007; Simon‐Bouhet et al., 2006).

In contrast to the nonsignificant *Φ*_ST_ indices, we furthermore found two of the oldest introductions, the clam *M*. *arenaria* and the crab *R*. *harrisii*, to be significantly differentiated by Jost's *D*. Hudson's Snn was even significant for all non‐natives but *T*. *navalis* and *H*. *takanoi* (the latter being the most recent introduction). This may indicate the beginning of observable differentiation, and further hints at limited connectivity of the North and Baltic Sea. Alternatively, the differentiated populations were founded by different introduction events, as has been suggested for the crab *R*. *harrisii* (Hegele‐Drywa et al., 2015). Overall, even the non‐native species do not contradict the limited gene flow we observed in native species.

### 4.3 Differentiation before the formation of the Baltic Sea

For four species, the cockle *Cerastoderma glaucum*, the amphipod *Gammarus locusta*, the shrimp *Palaemon varians*, and the copepod *Eurytemora affinis*, coalescent estimates dated the divergence of North and Baltic Sea populations in the Late Pleistocene between 200,000 and 450,000 years ago, often with much wider confidence intervals, but never including 8000 years. Two other species, the barnacle *Balanus crenatus* and the gastrotrich *Turbanella hyalina*, show similar genetic divergence patterns (Figure 5), but have insufficient data to generate robust coalescent estimates. Assuming similar divergence rates, Baltic Sea populations of these six species diverged from the North Sea populations much earlier than 8000 years ago. As the Baltic Sea to its current extent did not exist prior to the LGM, the divergence must have occurred somewhere else. For the cockle *C*. *glaucum*, long‐distance dispersal from the Iberian Peninsula to the Baltic Sea aided by migrating birds has been implied based on phylogeographic reconstructions (Tarnowska et al., 2010). For the copepod *E*. *affinis*, this much older divergence likely dates to a previous interglacial period in today's North Sea and East Atlantic (Remerie et al., 2009; Winkler et al., 2011). During the Pleistocene, the British Isles were connected to the European continent with a land bridge, which separated the ancient North Sea from the southern English Channel (Cohen et al., 2017). During the subsequent glaciation of both the Baltic Sea and North Sea, the respective populations must have retreated into separate glacial refugia. The land bridge between the British Isles and Europe remained ice‐free, and separated the Scandinavian and the British ice sheets due to much lower sea levels (Dawson, 1992). Marine organisms such as the amphipod *G*. *locusta*, the shrimp *P*. *varians*, the barnacle *B*. *crenatus*, and the gastrotrich *T*. *hyalina* may have retreated into glacial refugia located either south of the permanent ice shields (Luttikhuizen et al., 2012; Remerie et al., 2009) or in the Irish Sea, around Scotland, and in the English Channel (Provan et al., 2005; Roman & Palumbi, 2004). This means that ¼ of the native species diverged and remained separate for much longer than the current brackish water Baltic Sea has been in existence.

### 4.4 Genetic diversity and population size

We found that genetic haplotype diversity and theta of Baltic Sea populations are lower in only half of the species, and nucleotide diversity, in only ⅓ of the species, contrary to a previous study that found the majority of Baltic Sea populations to be less diverse (Johannesson & André, 2006). Six of those species, that is, the barnacle *Balanus crenatus*, the amphipods *Gammarus duebeni* and *G. zaddachi*, the mysid *Neomysis integer*, the gastrotrich *Turbanella hyalina*, and the cockle *Cerastoderma glaucum*, are also significantly differentiated from the North Sea. In these species, it seems likely that the Baltic Sea populations are self‐sustained but smaller than the North Sea populations, which is a hypothesis proposed earlier for Baltic Sea populations (Johannesson & André, 2006). The coalescent population size estimates, however, do not differ between North and Baltic Sea for *C. glaucum*. A population sink scenario, which predicts that the Baltic Sea population is not self‐sustained but replenished by propagules from the North Sea, also predicts lower genetic diversity in the Baltic Sea but no genetic differentiation between both populations. Genetically, this appears to be the case for six weakly differentiated species, that is, the echinoderms *Asterias rubens* and *Ophiura albida*, the shrimp *Palaemon elegans*, and the non‐natives *Rhithropanopeus harrisii*, *Eriocheir sinensis*, and *Acartia tonsa*. However, the echinoderms are adapted to the lower salinity conditions of the Baltic Sea, which contradicts the sink scenario. For the non‐natives, the sink pattern is a consequence of their recent colonization of the Baltic Sea, but does not reflect long‐term conditions. In other words, not enough time has passed to identify a potential lack of gene flow after the colonization event. Moreover, they remain largely untested for rapid adaptations to the lower salinity of the Baltic Sea. Only for the shrimp *Palaemon elegans*, the Baltic Sea may represent a true sink with lower genetic diversity and no genetic differentiation. That said, both Hudson's Snn and Jost's *D* differentiated the North and Baltic Sea populations of this species, which makes us wonder whether this species may not have adapted to the Baltic Sea after all. The introduction of the highly divergent Black Sea lineage of *P*. *elegans* further complicates the issue. While we could clearly identify and exclude sequences belonging to the Black Sea lineage, it is unclear whether the Atlantic lineage we considered here and the Black Sea lineage hybridize, and what the consequences may be for the Atlantic lineage. The coalescent estimates echo the finding that the Baltic Sea cannot be regarded as a population sink. Though several of the estimates had very wide bounds and did not converge properly, which was likely driven by insufficient data given large effective population sizes, the estimates did not reveal a consistent pattern. Migration rates were higher from the North Sea to the Baltic Sea than vice versa for *C*. *glaucum* and *G*. *locusta*, in line with a population sink scenario. However, neither species had smaller population size estimates for the Baltic Sea population. For *E*. *affinis* and *P*. *varians*, migration rates were even higher from the Baltic Sea to the North Sea, contradicting the sink hypothesis altogether.

### 4.5 Life history and salinity adaptations

The fact that species with a long PLD are limited to more saline waters is intriguing. It is compatible with the fact that the larval phase is often most sensitive to environmental conditions (Sherman et al., 2016). If the planktonic larval phase is shorter, or completely absent, this may increase the probability of a species to colonize brackish to freshwater environments. This theory is well supported by marine taxa that colonized rivers and freshwater by abbreviating or eliminating the planktonic larval phase (Vogt, 2013).

Dispersal ability did not correlate with population differentiation. This further strengthens our argument that limited water flow is not responsible for population differentiation. This mirrors results of comparative phylogeographic studies along, for example, the North and South American coasts, where dispersal ability is a poor predictor of population differentiation (Kelly & Palumbi, 2010). Instead, our results corroborate the idea that local adaptation drove population differentiation, in combination with small founding populations (Johannesson & André, 2006). Many species colonized the Baltic Sea early on, when the salinity was higher and the connectivity to the North Sea was stronger (Johannesson et al., 2011). Subsequent adaptations to declining salinities would have isolated the populations, which was exacerbated by decreasing North Sea water inflow (which is linked to the lowered salinity).

The basin‐specific differences in salinity tolerance are likely due to local adaptation. The experiments from which we derived the salinity tolerances do not allow us to infer the underlying evolutionary mechanism, which could be mutations or allele frequency changes of the genomic sequence, epigenetic changes, or acclimatization. Disentangling these effects will take multigenerational experiments in combination with detailed molecular approaches, but could generate unprecedented insight into the rapid evolution of freshwater tolerance. For example, long‐term common garden experiments followed by proteomics revealed several functional candidate loci that were differentially expressed between freshwater and brackish water spawning whitefish (Papakostas et al., 2012). These results suggest molecular adaptation to the respective salinity rather than acclimatization.

### 4.6 What do different ecological–genetic patterns indicate?

We identified species where genetics and ecology match up, either because both are differentiated across the North Sea–Baltic Sea gradient (7 spp.: *Cyanea capillata*, *Balanus crenatus*, *Gammarus locusta*, *G*. *oceanicus*, *Idotea balthica*, *Palaemon varians*, and *Turbanella hyalina*) or because both suggest homogeneity (4 spp.: *Acartia tonsa*, *Gammarus salinus*, *Palaemon elegans*, and *Mya arenaria*) (Figure 9). We also identified species with intermediate patterns, where either ecology (5 spp.: *Aurelia* sp., *Carcinus maenas*, all 3 investigated Echinodermata spp.) or genetics alone suggest differentiation (3 spp.: *Eurytemora affinis*, *Gammarus duebeni*, and *G*. *zaddachi*). For the remaining 9 species, including all non‐native species, comparative salinity tolerance estimates do not exist (Figure 9).

Ecological differentiation and local adaptation can occur within decades to centuries, for example, cold adaptation of the invasive Burmese Python in Florida (Card et al., 2018), habitat and diet shift of mangrove tree crabs in Georgia (Riley et al., 2014), or adaptation of mice to urban habitats (Harris et al., 2013). Thus, we may expect salinity adaptation to occur rapidly and frequently, a view borne out by our data. Genetic divergence of a putatively neutral marker, such as mitochondrial DNA, occurs much slower, and only when gene flow is severely limited (Messer et al., 2016).

The concordant differentiation of ecology and genetics across the North Sea–Baltic Sea gradient can be attributed, on the one hand, to a much older divergence, as in *B*. *crenatus*, *T*. *hyalina*, and *P*. *varians*. For other species that are less, but nonetheless significantly, differentiated, such as the amphipods *G*. *locusta* and *G*. *oceanicus*, or the isopod *I*. *balthica*, a divergence since the formation of the Baltic Sea is plausible. In these cases, their short generation times (Kolding & Fenchel, 1979; Leidenberger et al., 2012) let differentiation processes take place relatively fast with regard to years.

In the investigated echinoderms, the jellyfish *Aurelia* sp. and the crab *Carcinus maenas*, the population genetics show no significant differentiation between North and Baltic Sea populations. The ecology, however, indicates that the Baltic Sea populations are adapted to lower salinity. This could mean that gene flow is ongoing, and the populations adapted in the face of gene flow (Tigano & Friesen, 2016). Particularly for *C*. *maenas*, this hypothesis is likely. This species is a known invader across the globe, and therefore transported frequently across oceans. Alternatively, the relatively long generation time of these species—they all need one to two years to mature (Crothers, 1967; Jackson, 2008; Nichols & Barker, 1984)—leads to slow divergence between populations, such that gene flow may not be ongoing, but the mitochondrial gene marker has not (yet) accumulated enough differences to show divergence. Investigating more invertebrates with long generation times should allow us to confirm these results, for example, the large conchs *Neptunea antiqua* and *Buccinum undatum*, and the hermit crab *Pagurus bernhardus*, which occur in both North and Baltic Sea (Zettler et al., 2018).

Only three species are genetically differentiated between North and Baltic Sea populations, but do not show signs of ecological differentiation: the copepod *E*. *nordmanni* and the amphipods *G*. *duebeni* and *G*. *zaddachi*. These species are found deep into the Baltic Sea and display wide salinity tolerances in both North and Baltic Sea. They indicate limited gene flow in lieu of adaptation, and further highlight that there is little connectivity into the Baltic Sea.

It is apparent that the diversity of ecological–genetic patterns in the genus *Gammarus* is highest compared with other taxonomic groups considered in our study. A potential explanation for this diversity is the different life histories of the five *Gammarus* species: While *G*. *duebeni*, *G*. *zaddachi*, and *G*. *salinus* are “true” brackish water species that occur in river deltas and other brackish habitats outside the Baltic Sea, *G*. *oceanicus* and *G*. *locusta* occur under (almost) fully marine conditions in the North Sea/Atlantic Ocean (den Hartog, 1964; Fenchel & Kolding, 1979; Gaston & Spicer, 2001). The latter two species therefore had to evolve wider salinity tolerances to inhabit the Baltic Sea. In line with their ecological differentiation, neutral genetic differentiation is also substantial, indicating comparatively long divergence times (this paper for *G*. *locusta*, Normant et al., 2005, for *G*. *oceanicus*). For *G*. *duebeni* and *G*. *zaddachi*, on the other hand, the significant genetic divergence between North and Baltic Sea populations does not align with differences in salinity tolerance, because even the North Sea populations tolerate near‐freshwater conditions. However, Kolding and Fenchel (1979) found differing reproductive traits in North and Baltic Sea populations of both species, which could hint at the beginning reproductive isolation between these populations. In contrast to the diversity in *Gammarus*, the congruent pattern we found in all three echinoderms might be a consequence of their common evolutionary origin as fully marine species, leading to similar adaptation processes to lower salinities during their colonization of the Baltic Sea.

For the three species displaying homogeneity at the genetic and ecological level (*A*. *tonsa*, *G*. *salinus*, and *P*. *elegans*), individuals may be swept into the Baltic Sea without forming reproducing populations. However, given the lack of gene flow between North and Baltic Sea which we and others observed for the majority of organisms (Johannesson & André, 2006; Sjöqvist et al., 2015) in combination with the limited oceanographic connectivity that has been modeled (Barz et al., 2006; Hordoir et al., 2013), this scenario appears unlikely to us. Moreover, these species are common in the Baltic Sea and not limited to the most western parts. Instead, this apparent homogeneity is attributable on the one hand to a recent colonization of the Baltic Sea of species with a wide salinity tolerance, that is, the non‐natives *A*. *tonsa* and *M*. *arenaria*. This may also be the case for the amphipod *G*. *salinus*, which we consider the only native species that displays neither genetic nor ecological differentiation. On the other hand, some genetic divergence may exist, as we assume for the shrimp *P*. *elegans*, which we found significantly differentiated by two of the differentiation indices, but not by *Φ*_ST_. Furthermore, the recent introduction of the highly divergent Black Sea lineage of *P*. *elegans* may complicate the assessment of salinity tolerance. This species clearly warrants further investigation, but for the moment, the Baltic Sea entrance may not be considered a barrier for this species.

In summary, our data provide evidence for the coexistence of divergent eco‐evolutionary trajectories in different marine invertebrate species that inhabit the North Sea–Baltic Sea region. These trajectories appear to be shaped by a complex interplay of the species’ ecology, evolutionary background, and colonization history (compare Ewers‐Saucedo & Wares, 2020). Future studies including both, additional life history traits and genomic/non‐neutral genetic markers could draw a more detailed picture of the formation of these trajectories.

### 4.7 The Baltic Sea: A natural experiment on the tempo and mode of adaptation

We show that the transition from North Sea to Baltic Sea represents a barrier to gene flow for most marine invertebrates, many of which have altered salinity tolerances in the Baltic Sea. This genetic and ecological divergence is congruent with similar findings for fishes and algae (Berg et al., 2015; Guo et al., 2015; Johannesson & André, 2006; Papakostas et al., 2012; Sjöqvist et al., 2015; Wennerström et al., 2013), which makes this gradient ubiquitous across phyla. The Baltic Sea provides the unique opportunity to understand the mechanisms underlying this differentiation, and particularly the mechanisms of salinity adaptation. Particularly, the combined use of non‐native and native species would allow a comparison of species at different stages of a colonization process that is almost certain to require adaptation, from ongoing colonization and expansion as in the crab *H*. *takanoi*, to recent colonization within the last few centuries as in in the mitten crab *E*. *sinensis*, or colonization many centuries ago as in the clam *M*. *arenaria* to several thousand years as in the amphipod *G*. *duebeni* and finally a divergence several hundred thousand years as in the barnacle *B*. *crenatus*. The physical proximity between the North and Baltic Sea facilitates ecological common garden experiments across a natural salinity gradient (Sjöqvist et al., 2015). With the emerging suite of genomic tools, such experiments advance our understanding of adaptation and colonization (Sherman et al., 2016). While they have been predominantly restricted to fishes (Larsen et al., 2008; Papakostas et al., 2012), our results highlight the great potential of transferring such approaches to additional (invertebrate) species to achieve a more complete understanding of the evolution of the Baltic Sea's unique species community.

## CONFLICT OF INTEREST

We declare no conflict of interest.

## AUTHOR CONTRIBUTIONS

**Jonas C. Geburzi:** Conceptualization (equal); Data curation (equal); Formal analysis (equal); Methodology (equal); Resources (equal); Visualization (equal); Writing – original draft (supporting); Writing – review & editing (equal). **Nele Heuer:** Data curation (equal); Investigation (supporting); Resources (supporting); Validation (supporting); Writing – review & editing (supporting). **Lena Homberger:** Data curation (equal); Investigation (supporting); Validation (supporting); Writing – review & editing (supporting). **Jana Kabus:** Data curation (equal); Investigation (supporting); Resources (supporting); Writing – review & editing (supporting). **Zoe Moesges:** Data curation (equal); Investigation (supporting); Resources (supporting); Validation (supporting); Writing – review & editing (supporting). **Kira Ovenbeck:** Data curation (supporting); Formal analysis (supporting); Investigation (supporting); Resources (equal); Writing – review & editing (supporting). **Dirk Brandis:** Conceptualization (equal); Funding acquisition (lead); Methodology (equal); Resources (equal); Supervision (equal); Writing – review & editing (supporting). **Christine Ewers:** Conceptualization (lead); Data curation (lead); Formal analysis (equal); Investigation (equal); Methodology (equal); Software (lead); Supervision (lead); Validation (equal); Visualization (equal); Writing – original draft (lead); Writing – review & editing (equal).

## Supporting information

Fig S1

Table S1

Table S2

## Data Availability

All new sequence data that have been used in this study have been uploaded to the NCBI GenBank (https://www.ncbi.nlm.nih.gov/genbank/); see Table S2 in the supporting information for accession numbers. The output files of the IMa2 software for coalescent time estimates and all R scripts for data analyses are available at https://doi.org/10.6084/m9.figshare.c.5341910.
