# Supplementary material for: An environmental gradient dominates ecological and genetic differentiation of marine invertebrates between the North and Baltic Sea
Source: Ecol Evol. 2022 May 20;12(5):e8868. doi: 10.1002/ece3.8868 (PMC9121054; doi:10.1002/ece3.8868)
Supplement: Supplementary file 1 — Fig S1 [file ECE3-12-e8868-s003.pdf]

Supporting Information 1 – Supplemental figures

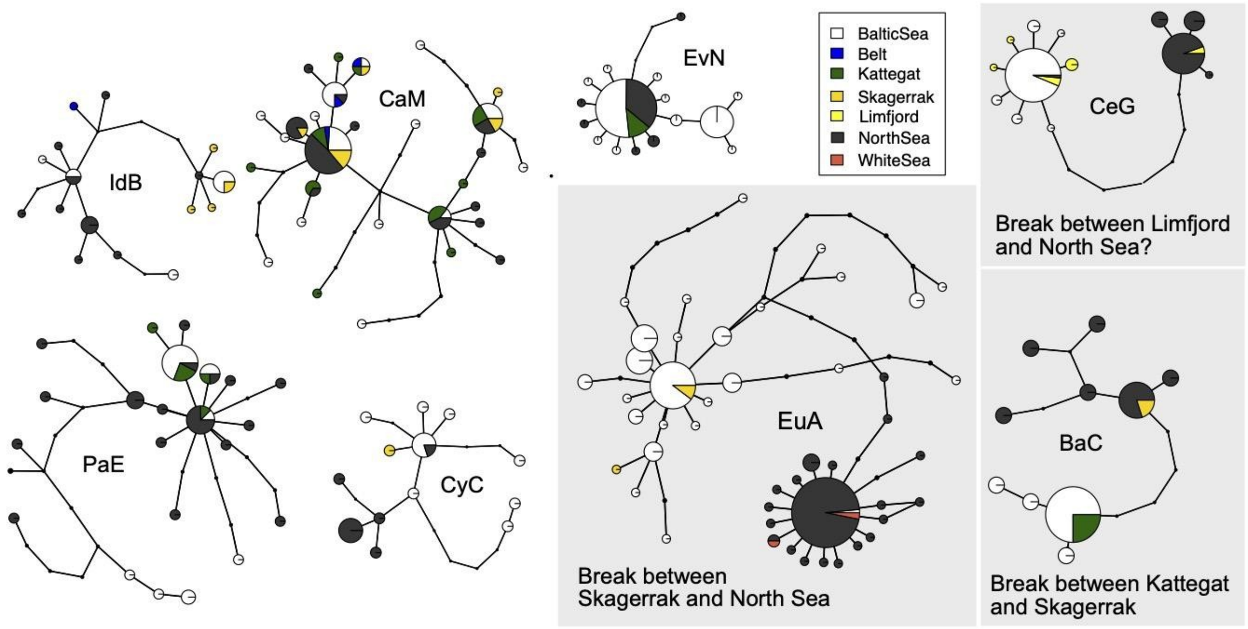

Figure S1.1. Extended haplotype networks for all species, for which sequences from the transition zone (as defined in Fig. 1 of the main manuscript) existed. The grey boxes indicate species with high differentiation between North and Baltic Sea populations, which allowed the inference of the phylogeographic break between these lineages. Abbreviations as in Tab. 1 of the main article.

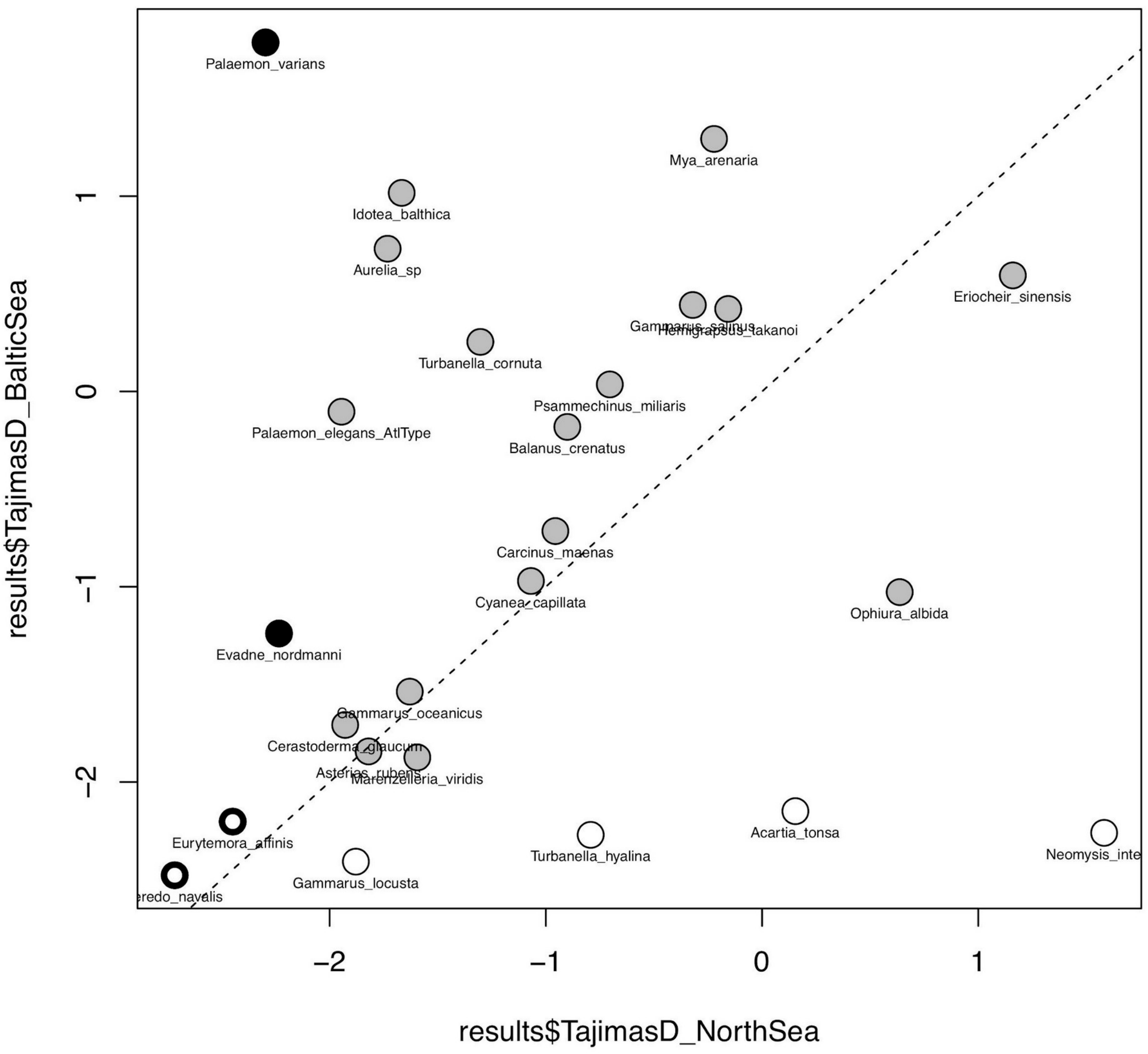

Figure S1.2. Estimates of Tajima's D. Grey: Tajima's D not significantly different from zero for either population, black: Tajima's D significantly different from zero in the North Sea population, white: Tajima's D significantly different from zero in the Baltic Sea population, and thick black circles: both populations significantly different from zero.
